# Sentinel lymph node biopsy omission in early-stage breast cancer: current evidence and clinical practice

**DOI:** 10.3389/fonc.2025.1598730

**Published:** 2025-06-19

**Authors:** Tao Huang, Wenkang Wang, Xianfu Sun

**Affiliations:** ^1^ Department of Breast Disease, Henan Breast Cancer Center, The Affiliated Cancer Hospital of Zhengzhou University & Henan Cancer Hospital, Zhengzhou, China; ^2^ Department of Breast Surgery, The First Affiliated Hospital of Zhengzhou University, Zhengzhou, China

**Keywords:** early-stage breast cancer (EBC), sentinel lymph node biopsy, SLNB omission, axillary management, de-escalation surgery

## Abstract

Recent trials (e.g., SOUND, INSEMA) indicate that omitting SLNB in patients with small tumors, negative imaging, and favorable prognoses maintains outcomes, reduces complications, and improves quality of life (3,4). Specifically, the INSEMA trial demonstrated that patients who omitted SLNB experienced significantly lower rates of persistent lymphedema (1.8% vs. 5.7%), restriction of arm/shoulder mobility (2.0% vs. 3.5%), and pain with arm/shoulder movement (2.0% vs. 4.2%) compared to those undergoing SLNB. Consequently, guidelines now suggest omitting routine SLNB in elderly patients and those with very low-risk tumors(2). This review summarizes current evidence, eligible populations, theoretical rationale, controversies, and future directions regarding SLNB omission in early breast cancer management.

## 1 Introduction

Since sentinel lymph node biopsy (SLNB) was introduced, breast cancer axillary management shifted from routine dissection to precise SLNB staging, reducing unnecessary surgeries (1). However, both axillary lymph node dissection (ALND) and even the less invasive SLNB can lead to postoperative complications such as pain, lymphedema, and restricted upper limb mobility, significantly impacting patients’ long-term quality of life. Therefore, minimizing or even omitting axillary surgery altogether, when oncologically safe, has become a crucial goal to further decrease surgical morbidity. Advances in systemic therapy, radiotherapy, and tumor biology understanding further support safely omitting SLNB in select early-stage patients (2).

Recent trials (e.g., SOUND, INSEMA) indicate that omitting SLNB in patients with small tumors, negative imaging, and favorable prognoses maintains outcomes, reduces complications, and improves quality of life (3, 4). Specifically, the INSEMA trial demonstrated that patients who omitted SLNB experienced significantly lower rates of persistent lymphedema (1.8% vs. 5.7%), restriction of arm/shoulder mobility (2.0% vs. 3.5%), and pain with arm/shoulder movement (2.0% vs. 4.2%) compared to those undergoing SLNB. Consequently, guidelines now suggest omitting routine SLNB in elderly patients and those with very low-risk tumors (2).

This review summarizes current evidence, eligible populations, theoretical rationale, controversies, and future directions regarding SLNB omission in early breast cancer management.

## 2 Theoretical basis for omitting axillary lymph node biopsy

### 2.1 Systemic therapy and radiotherapy as effective means of controlling minimal residual disease

Systemic therapies, including chemotherapy, targeted agents, endocrine treatments, and immunotherapies, have demonstrated substantial efficacy in eliminating microscopic disease that persists beyond surgical intervention, particularly within axillary regions (5–7). These therapies significantly contribute to lowering recurrence and metastatic potential, thus reinforcing the rationale for axillary surgery reduction.

Radiotherapy similarly provides robust control over subclinical axillary involvement through several distinct mechanisms. Direct cytotoxicity is achieved through regional lymph node irradiation, including axillary and supraclavicular nodes, delivering lethal doses that induce DNA damage and subsequent tumor cell apoptosis. Additionally, incidental irradiation during whole-breast or chest wall radiotherapy often encompasses portions of the axillary level I nodes, imparting approximately 20–30 Gy (8), sufficient to markedly decrease regional recurrence compared to mastectomy without adjuvant radiotherapy (9). Moreover, radiotherapy promotes immunogenic cell death, thereby facilitating antigen release and dendritic cell activation, ultimately enhancing systemic anti-tumor immunity (10). Consequently, radiotherapy not only directly eradicates axillary tumor cells but also alters the tumor microenvironment, reducing immune suppressive cell populations and increasing cytotoxic T-cell infiltration. These combined effects provide a robust biological justification for the strategic de-escalation of axillary surgical interventions.

### 2.2 Insights from key clinical trials on surgical de-escalation in small-volume axillary metastases

Recent pivotal clinical trials have demonstrated that omission of axillary lymph node dissection (ALND) in patients with limited sentinel lymph node (SLN) involvement (1–2 positive nodes) does not adversely affect recurrence or survival outcomes, provided comprehensive systemic therapy and radiotherapy are employed (11–13). Landmark trials such as ACOSOG Z0011 (11) (11% residual positivity), IBCSG 23-01 (12) (13%), and AMAROS (13) (up to 33%) collectively indicate that minimal residual axillary tumor burden can be effectively controlled through subsequent radiotherapy and systemic therapies. These results underpin the rationale for considering the omission of SLNB in carefully selected patient populations.

### 2.3 Shifting paradigm: biological risk stratification in axillary management

The contemporary approach to axillary staging in breast cancer management increasingly relies on biological and genomic risk assessments rather than exclusively on nodal pathology (14).

Triple-negative breast cancer (TNBC) and HER2-positive breast cancer, both characterized by higher aggressiveness, have systemic treatments highly dependent on tumor biology rather than axillary staging. Current clinical guidelines consistently recommend systemic chemotherapy ± targeted or immunotherapy for these two breast cancer subtypes if the tumor diameter is ≥2 cm (T2 or greater) or when high-risk biological features are present, regardless of clinically and radiologically assessed node-negative status (N0) (15). Consequently, the presence or absence of axillary lymph node metastasis does not alter the established therapeutic regimen.T1-stage breast cancers have low axillary lymph node metastasis rates, SEER data show rates of 1.42% for T1a, 2.26% for T1b, and 6.12% for T1c (16). Therefore, if SLNB is omitted, the risk of undertreatment is low, especially when preoperative imaging rules out obvious nodal disease or suspicious nodes are biopsied. Still, for T1-stage TNBC and HER2-positive cases, any decision to omit SLNB should be made with careful, individualized risk assessment and MDT discussion.

Furthermore, genomic assays such as Oncotype DX and the 70-gene signature significantly influence chemotherapy decision-making in hormone receptor-positive patients with limited axillary disease, largely independent of precise nodal status (17, 18). Consequently, precise axillary node status determination may not substantially impact clinical management in patients with aggressive tumor biology or very favorable prognostic profiles. This shift highlights a diminishing clinical necessity for SLNB, supporting its safe omission in appropriately selected cases.

In summary, these evolving theoretical and clinical insights underscore the decreasing therapeutic relevance of axillary lymph node biopsy for pathological staging. Undetected minimal axillary disease can be adequately managed through adjunctive treatments, thereby preserving survival and local disease control. This framework constitutes the essential theoretical basis justifying the omission of SLNB.

## 3 Alternative or adjunctive methods for axillary evaluation

Given the evolving evidence supporting SLNB omission, clinical interest has increasingly focused on non-invasive diagnostic methods to accurately identify patients with minimal or absent axillary metastatic disease.

### 3.1 Imaging modalities

Ultrasound (US) remains one of the most commonly utilized imaging techniques for preoperative axillary evaluation, notable for its high specificity yet moderate sensitivity. A meta-analysis (19) reported US sensitivity for detecting axillary metastases as approximately 50–60%, corresponding to a false-negative rate of 40–50%, while specificity was exceptionally high (97–100%). Consequently, although suspicious ultrasound findings carry high positive predictive value, about half of small metastatic lesions may go undetected. Breast and axillary MRI (20), with slightly lower specificity (90–100%), occasionally misclassifies benign nodes as malignant but offers improved sensitivity (73–94.6%) compared to ultrasound, enhancing detection rates for small metastatic deposits despite persistent false negatives. Thoracic CT, not primarily intended for axillary staging, reliably detects larger lymph node metastases but exhibits limited sensitivity for smaller lesions, reflected by a false-negative rate of 12–28% (21, 22). PET-CT (23) demonstrates excellent sensitivity for larger metastases but remains insensitive to smaller lesions, with approximately 34% false-negative findings. Thus far, no imaging modality achieves sufficient diagnostic precision to entirely replace invasive axillary staging.

### 3.2 Molecular detection of cfDNA and ctDNA

In recent years, circulating cell-free DNA (cfDNA) in peripheral blood has shown promising potential for predicting axillary lymph node (ALN) metastasis in breast cancer. A meta-analysis involving 5,736 patients demonstrated a significant association between elevated cfDNA levels and ALN metastasis (OR ≈ 2.15, P = 0.03) (24). However, the heterogeneity in detection platforms and methods across existing studies underscores the urgent need for standardization and harmonization to enable clinical translation. Additionally, circulating tumor DNA (ctDNA), despite showing strong predictive performance in disease recurrence (OR ≈ 3.79) and prognosis (DFS HR ≈ 5.18, OS HR ≈ 2.43), has demonstrated insufficient sensitivity for predicting ALN metastasis (OR ≈ 1.76, P = 0.11), making it more suitable for postoperative monitoring and long-term prognosis rather than initial ALN assessment. Studies have also indicated that patients with persistent ctDNA after surgery have a significantly higher incidence of ALN metastasis, although this finding is mainly relevant to detecting minimal residual disease (MRD) postoperatively and does not provide effective preoperative ALN prediction (25).

Recently, a study conducted low-depth whole-genome sequencing on plasma samples from 330 breast cancer patients and developed a cfDNA promoter-fragment profiling classification model named “PPCNM” using machine learning algorithms (26). This model achieved an AUC of 0.897, with sensitivity and specificity of 0.914 and 0.881 respectively. This was the first demonstration that the cfDNA fragment coverage patterns at gene promoter regions could reflect tumor aggressiveness and metastatic potential, highlighting cfDNA fragmentomics as a promising non-invasive tool for ALN staging. Further research should prioritize standardization of detection methods and validation through large-scale, multicenter studies to facilitate clinical adoption.

Whereas conventional imaging modalities delineate the anatomical extent and spatial distribution of nodal metastases, molecular assays offer orthogonal, biology-based insights that reflect tumor burden and dissemination potential. An integrated diagnostic paradigm combining both structural and molecular data may substantially refine axillary staging strategies. In clinical scenarios marked by equivocal imaging findings, concordant positivity on molecular testing could bolster diagnostic confidence for nodal involvement. Conversely, the simultaneous absence of radiologic and molecular abnormalities may support the safe de-escalation of axillary intervention, including potential omission of SLNB, thereby advancing a precision-tailored, minimally invasive therapeutic approach.

## 4 Clinical trials and evidence-based insights: from SLNB reduction to SLNB omission

### 4.1 Early clinical trials on axillary surgical de-escalation: Z0011, IBCSG 23-01, and AMAROS

- ACOSOG Z0011 Trial (11): This seminal study enrolled women with clinical stage T1–2 invasive breast cancer (tumors ≤5 cm), no palpable axillary adenopathy (cN0), and planned for breast-conserving surgery (lumpectomy) followed by tangential whole-breast irradiation. Patients received no prior systemic therapy. Eligible patients had metastases detected by H&E stain in only 1 or 2 sentinel lymph nodes (SLNs) and were randomized to SLND alone versus completion axillary lymph node dissection (ALND). The trial demonstrated no significant differences in regional recurrence, disease-free survival, or overall survival between the two groups. Hence, patients meeting Z0011 eligibility criteria could safely omit further ALND without adversely affecting oncological outcomes. The Z0011 findings established the basis for axillary surgical de-escalation, substantiated by extensive follow-up and subsequent validation studies.
- IBCSG 23–01 Trial (12): Conducted across multiple international centers, this trial specifically targeted breast cancer patients with tumors ≤5 cm and clinically negative axillary nodes (cN0) who, after SLNB, were found to have only micrometastases (≤2 mm, including isolated tumor cells) in one or more sentinel nodes, with no extracapsular extension. Patients were randomized to ALND or no further axillary surgery. Similar to Z0011, this trial demonstrated that omitting ALND in patients with minimal sentinel node involvement did not increase the risk of recurrence or mortality. Although SLNB was still utilized, the outcomes indicated that limited axillary metastatic disease might not necessitate further axillary surgery, implicitly supporting consideration of completely omitting SLNB in highly selected scenarios.
- AMAROS Trial (13): This randomized study enrolled patients with T1–2 primary, invasive breast cancer (initially ≤3 cm, later amended to ≤5 cm or multifocal) and no palpable lymphadenopathy (cN0). Patients underwent SLNB, and those found to have a positive sentinel node (macrometastasis, micrometastasis, or initially isolated tumor cells before amendment) were randomized to receive either ALND or axillary radiotherapy. Results revealed equivalent outcomes in regional control and recurrence rates, positioning radiotherapy as a viable alternative to surgical lymph node clearance with the added benefit of reduced morbidity (significantly less lymphedema). Thus, AMAROS reinforced the feasibility of substituting invasive axillary surgical interventions with radiotherapy, especially in cases of limited nodal involvement.

Collectively, although these trials did not directly investigate the complete omission of SLNB, their results strongly indicate that minimal axillary metastatic disease can be effectively managed through adjuvant therapies, such as radiotherapy and systemic treatment, thereby reducing the necessity for extensive surgical intervention.

### 4.2 Contemporary SLNB omission trials: SOUND and INSEMA

The SOUND trial (3) is among the initial randomized controlled studies evaluating the complete omission of SLNB. The trial enrolled 1,405 patients with early-stage breast cancer (tumor ≤2 cm), clinically node-negative axilla, and normal axillary ultrasound findings. Participants were randomized into either a no-axillary-surgery group (n=697) or a standard SLNB group (n=708), with all patients undergoing breast-conserving surgery followed by radiotherapy. At a median follow-up, the 5-year distant disease-free survival (DDFS) was non-inferior in the no-SLNB group (98.3%) compared to the SLNB group (98.7%), with no statistically significant difference (P=0.67; HR=0.84, 90% CI: 0.45-1.54; non-inferiority P=0.02). SLNB identified axillary metastases in 13.7% of patients (5.1% micrometastases and 8.6% macrometastases). Despite a similar estimated prevalence of undetected nodal metastases in the omission group, axillary recurrence remained very low (0.4%). The postoperative systemic treatments (endocrine therapy, chemotherapy, radiotherapy) were comparable across both groups, indicating no apparent undertreatment due to SLNB omission. Additionally, the 5-year overall survival rates were similar (98.2% without SLNB vs. 98.2% with SLNB). Investigators concluded that SLNB omission is safe for selected patients, particularly those with small tumors and negative axillary imaging, provided the nodal status does not influence therapeutic decisions. However, they advised careful multidisciplinary assessment for younger patients whose nodal status might affect treatment choices, such as chemotherapy decisions.

The INSEMA trial (4), a larger European randomized study, involved over 5500 clinically node-negative (T1-2N0) breast cancer patients, predominantly characterized as T1 (90%), HR-positive/HER2-negative (95%), and aged ≥50 years (90%). Patients were randomized between standard SLNB and complete axillary surgery omission. At approximately six years median follow-up, the 5-year invasive disease-free survival (iDFS) rates were nearly identical (91.9% without SLNB vs. 91.7% with SLNB; HR=0.91), meeting non-inferiority criteria. Axillary recurrence rates were marginally higher in the no-SLNB group (1.0% vs. 0.3%), but this difference was minor. Importantly, omitting SLNB significantly improved quality-of-life outcomes, reducing rates of lymphedema, enhancing arm mobility, and decreasing symptoms such as pain and swelling. Investigators highlighted that older patients or those with HR-positive/HER2-negative disease profiles are optimal candidates for SLNB omission, though additional data is needed for tumors larger than 2 cm.

Other ongoing trials—including BOOG 2013-08 (27) in Europe, NAUTILUS (28) in South Korea, and the single-arm SOAPET (29) trial in China—are similarly assessing the feasibility of safely omitting SLNB through advanced imaging techniques in clinically node-negative early breast cancer patients. Findings from these studies are expected to further refine patient selection and potentially broaden clinical applicability of SLNB omission.

## 5 Guideline recommendations for SLNB exemption

Recent guidelines from major global breast cancer organizations increasingly support omitting SLNB for elderly or low-risk individuals. For instance, the 2021 Ontario Health (Cancer Care Ontario) and ASCO guidelines (30) explicitly recommend against routine SLNB in patients aged ≥70 years with clinical T1N0, hormone receptor (HR)-positive, HER2-negative early-stage invasive breast cancer. These guidelines further emphasize preoperative consultation with medical oncologists to ensure appropriate systemic therapy planning if SLNB is omitted.

The German AGO guidelines (31) also support optional omission of SLNB for elderly or comorbid patients with favorable tumor biology (≥70 years, T1N0, HR-positive/HER2-negative).

Likewise, the National Comprehensive Cancer Network (NCCN) guidelines (15) state that the performance of axillary staging may be considered optional for patients who have particularly favorable tumors, those for whom adjuvant systemic and/or radiotherapy selection is unlikely to be impacted, elderly individuals, or those with serious comorbid conditions.

In summary, the international guidelines increasingly recognize and support SLNB omission in carefully selected low-risk patients, underscoring the necessity of patient-centered communication and multidisciplinary assessment to guarantee comprehensive systemic or radiotherapy coverage for potentially undetected minimal metastases.

## 6 Controversies and challenges

A primary controversy regarding SLNB omission is the potential loss of prognostic information, possibly affecting subsequent treatment decisions. Some experts argue that axillary staging does not significantly alter treatment strategies in low-risk patients. Conversely, others are concerned that nodal positivity—even among those initially not considered candidates for chemotherapy—could influence additional therapeutic decisions, such as extending endocrine therapy to ten years or incorporating CDK4/6 inhibitors for hormone receptor-positive patients. This concern is particularly relevant for younger patients, where positive lymph nodes often necessitate more aggressive chemotherapy, and completely omitting axillary assessment could lead to undertreatment. In response to data from trials like SOUND and INSEMA, Professor Monica Morrow have proposed a compromise strategy: initially omitting SLNB in low-risk patients and performing delayed axillary surgery only when unexpected high-risk pathological features emerge postoperatively (e.g., tumor upgrading to T2, grade 3, or lymphovascular invasion) (32). The feasibility and safety of this “delayed rescue” approach require validation in clinical studies.

Another significant debate involves radiotherapy to the axilla following SLNB omission. Traditionally, elderly patients with confirmed node-negative status and low-risk tumors could safely forgo postoperative breast irradiation (33). However, when axillary status is uncertain due to omitted SLNB, clinicians may prefer comprehensive breast and axillary irradiation as a precautionary measure. In the SOUND trial (3), nearly all patients (>97%) received radiotherapy, potentially increasing treatment burden for older patients who might have otherwise avoided radiation. Consequently, the clinical trade-off between reduced surgical intervention and potentially increased radiotherapy is a critical discussion point.

## 7 Summary

The publication of recent prospective randomized trials, notably SOUND and INSEMA, has transitioned the omission of SLNB in breast cancer from a theoretical concept to an evidence-supported clinical practice. Current robust evidence indicates that omitting SLNB in carefully selected patients with tumors ≤2 cm, hormone receptor-positive, HER2-negative, and clinically as well as radiologically negative axillary lymph nodes does not negatively impact oncologic outcomes. Furthermore, this strategy significantly decreases surgical morbidity, particularly reducing complications such as upper limb lymphedema, thereby substantially improving patient quality of life. Advances in diagnostic imaging and molecular profiling technologies are anticipated to further extend eligibility for SLNB omission to broader populations of low-risk breast cancer patients. Such advancements promise to optimize personalized treatment approaches, minimize therapeutic invasiveness, and maintain excellent long-term oncologic safety and axillary control.
